# Coronary artery bypass grafting using both internal mammary arteries—a safe concept for surgical training

**DOI:** 10.1093/icvts/ivaf100

**Published:** 2025-04-25

**Authors:** Tim Knochenhauer, Yvonne Schneeberger, Martin Beyer, Friedrich Sobik, Xiaoqin Hua, Beate Reiter, Jens Brickwedel, Svante Zipfel, Hermann Reichenspurner, Lenard Conradi, Bjoern Sill, Andreas Schaefer

**Affiliations:** Department of Cardiovascular Surgery, University Heart and Vascular Center Hamburg, Hamburg, Germany; Department of Cardiovascular Surgery, University Heart and Vascular Center Hamburg, Hamburg, Germany; Department of Cardiovascular Surgery, University Heart and Vascular Center Hamburg, Hamburg, Germany; Department of Cardiovascular Surgery, University Heart and Vascular Center Hamburg, Hamburg, Germany; Department of Cardiovascular Surgery, University Heart and Vascular Center Hamburg, Hamburg, Germany; Department of Cardiovascular Surgery, University Heart and Vascular Center Hamburg, Hamburg, Germany; Department of Cardiovascular Surgery, University Heart and Vascular Center Hamburg, Hamburg, Germany; Department of Cardiovascular Surgery, University Heart and Vascular Center Hamburg, Hamburg, Germany; Department of Cardiovascular Surgery, University Heart and Vascular Center Hamburg, Hamburg, Germany; Department of Cardiovascular Surgery, University Heart and Vascular Center Hamburg, Hamburg, Germany; Department of Cardiovascular Surgery, University Heart and Vascular Center Hamburg, Hamburg, Germany; Department of Cardiovascular Surgery, University Heart and Vascular Center Hamburg, Hamburg, Germany

**Keywords:** CABG, surgical training, complete arterial revascularization, BIMA, education

## Abstract

**OBJECTIVES:**

In our centre, a bilateral internal mammary artery first approach is established from day 1 of surgical training. We herein aimed to investigate safety and clinical efficacy of this training concept.

**METHODS:**

All patients undergoing isolated bypass grafting between 2009 and 2021 at our institution were included in this study. Patients provided with single mammary artery, radial artery or vein grafts were excluded. According to a preoperative evaluation conducted by experienced coronary bypass surgeons, coronary artery disease severity was classified, and patients were allocated to group 1 (surgery performed by staff surgeons) and group 2 (surgery performed by residents under supervision of staff surgeons). Thirty-day outcome parameters were compared between groups.

**RESULTS:**

A total of 2125 patients were allocated to group 1, and 431 patients were assigned to group 2. Patients in group 1 presented a higher risk profile. Coronary artery bypass grafting in group 2 was more often performed as on-pump procedure with a longer procedure duration. Number of performed bypasses was lower in group 2 with fewer composite grafting and fewer anastomoses to the RCA territory. No significant differences in 30-day all-cause mortality, myocardial infarction, stroke or acute kidney injury were seen.

**CONCLUSIONS:**

Thirty-day outcomes after coronary artery bypass grafting using bilateral internal mammary artery grafts performed by residents were without significant differences to outcomes of staff surgeons, suggesting that application of both internal mammary arteries by residents is safe and effective when performed under supervision and after preoperative patient screening.

## Graphical Abstract

**Figure:**
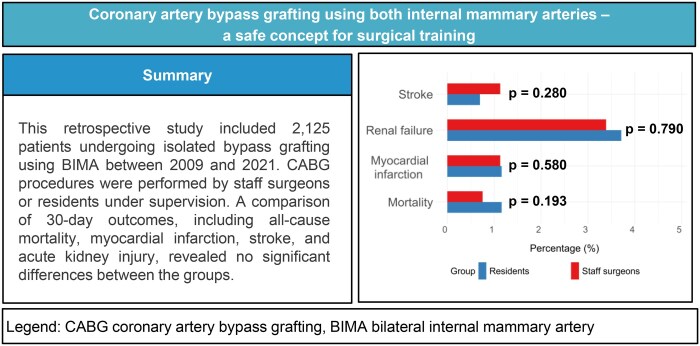

## INTRODUCTION

According to international guidelines, coronary artery bypass grafting (CABG) is the therapy of choice for patients with complex and multivessel coronary artery disease (CAD) [1, 2]. Over the last decades, different bypass grafting techniques and bypass grafts for CABG were described. Current evidence shows that grafting of the left internal mammary artery (LIMA) to the left anterior descending (LAD) presents excellent long-term results [3]. However, although a second arterial graft (right internal mammary artery [RIMA], radial artery [RA]) presents superiority in long-term graft patency compared to saphenous vein grafts (SVGs) [4–6], there is still a widespread utilization of SVG in CABG [7]. This may be attributable to the fact that complete arterial revascularization demands complex surgical techniques such as composite and sequential grafting, demanding a higher standard of surgical expertise [8].

Since complete arterial revascularization for CAD is recommended by international guidelines, it is of paramount importance to apply these techniques early in cardiac surgery training, to provide best patient care and build surgical capabilities for complex CABG. Besides available simulation-based models for anastomosis training with satisfying results in terms of suturing improvement [9–12], adoption of complete arterial revascularization in early steps of CABG training may be essential to increase utilization of a second arterial graft. At the authors' institution, all residents perform CABG using two arterial grafts with an aortic no-touch technique from day 1 of surgical training when anatomically and patient specific suitable. We herein aimed to compare outcomes of residents and experienced surgeons in CABG using both internal mammary arteries (bilateral internal mammary artery [BIMA]) to investigate whether this early adoption of complex CABG techniques influences outcomes of patients or the clinical efficacy of CABG.

## MATERIALS AND METHODS

### Patients and definitions

All patients undergoing isolated CABG between 2009 and 2021 at our institution were consecutively included in this study. Inclusion criteria were BIMA use, age >18 years and informed consent. Analysis included elective and emergency cases. Exclusion criteria were CABG procedures with single mammary artery, radial artery or vein grafts. There were no specific exclusion criteria for patients’ comorbidities.

**Figure 1:**
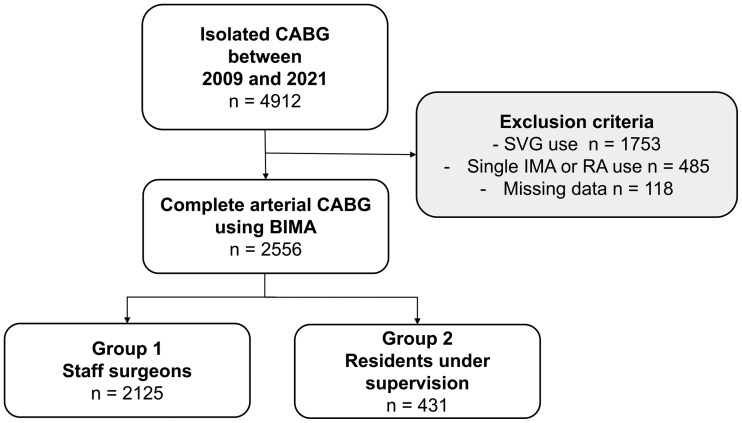
Patient inclusion flowchart. All patients undergoing isolated CABG between 2009 and 2021 at our institution were consecutively included in this study. Patients with a single arterial graft, alternative arterial grafts other than internal mammary arteries, vein grafts, or aortic insertion of the graft were excluded. CABG: coronary artery bypass grafting; BIMA: bilateral internal mammary artery; SVG: saphenous vein graft; IMA: internal mammary artery

Allocation of patients to staff surgeons or residents was conducted by ‘eyeballing’ of coronary angiographies by experienced surgeons in CABG including factors such as anticipated numbers of required distal bypasses and coronary anatomy (calcification, diameter of coronary arteries). Furthermore, clinical status of the respective patient was taken into consideration. A patient inclusion flowchart is shown in Fig. 1.

Data acquisition was performed anonymized and retrospectively. In accordance with German law (§12 HmbKHG), no ethics committee approval was needed for this study, and informed patient consent was waived. The analysis was conducted according to the Declaration of Helsinki (as revised in 2013).

Primary outcome parameters for this study are all-cause mortality, postoperative myocardial infarction, major stroke and renal failure (KDIGO ≥ 1; Kidney Disease: Improving Global Outcomes). All examined outcomes are 30-day outcomes.

Secondary outcomes included postoperative mechanical circulatory support, prolonged ventilation and use of catecholamines, hospital stay, intensive care unit (ICU) stay, wound infections and deep sternal wound infections (DSWIs).

Complete revascularization was defined as revascularization of all coronary segments with a stenosis of ≥ 50% supplying viable myocardium. DSWI was defined as postoperative infection involving the sternum and mediastinal space.

### Diagnostic work-up and study procedure

The preprocedural diagnostic work-up followed institutional standards: by routine, all patients received preoperative coronary angiography and transthoracic echocardiography (TTE) as well as X-ray of the chest and ultrasound examination of the carotid arteries. Viability of myocardium is not determined as part of the standard preoperative evaluation. However, viability is determined by magnetic resonance imaging when patients present with an occluded coronary artery with according akinesis of the provided myocardium. In our clinic, it is standard practice to perform preoperative duplex sonographic imaging of the carotid arteries for patients with risk factors for peripheral artery disease or an existing condition. Surgery was carried out under general anaesthesia and ventilation via full sternotomy and a transoesophageal probe was placed. All patients received a single shot of intravenous cefuroxime as standard perioperative antibiotic prophylaxis during the study period, provided there were no relevant allergies. Mammary arteries were harvested in a skeletonized fashion.

### Surgical education

General regulations for surgical education are given by the local medical association. At our institution, a stepwise educational programme was established. The first 3 years of training include ward rounds, outpatient department, ICU rotation as well as training of basic cardiac surgery techniques (sternotomy, establishment of cardiopulmonary bypass, harvesting of BIMA). Furthermore, a wet- and dry-lab curriculum was established, to improve surgical skills during the entire residency. Dry-lab facility, equipped with models for practicing bypass procedures and general handling of surgical instruments and sutures, is freely accessible at all times and is used under personal responsibility. Wet-lab exercises are conducted in small groups approximately 6–8 times per year and are supervised by experienced staff surgeons.

The dedicated cardiac surgery training starts in the fourth year of residency including a mentor-mentee team model. As part of the mentor-mentee programme, the residents begin by assisting in bypass surgeries and are gradually guided by the mentor towards independent performance of the procedure. After the mentor’s assessment, the residents perform the surgery independently, under the supervision of staff surgeons. Complete arterial revascularization using BIMA is the standard from the beginning of CABG training and is performed under supervision of CABG specialists.

### Statistics

Baseline, intraprocedural and acute follow-up data up to 30 days were retrospectively collected and entered into a standardized database.

Complete case analysis was performed, and cases with missing data were excluded from the analysis.

Influence of annual surgeon CABG procedure volume on postoperative outcome was assessed by using restricted cubic spline (RCS) transformation of the procedure volume.

To monitor and detect changes in postoperative mortality over cases, a cumulative sum (CUSUM) analysis was performed for each surgeon. Reference value was the patient’s individual EuroSCORE II. CUSUM control limits were set based on a 2-sigma approach (2 standard deviations from the expected mean), and were uniformly applied to all surgeons.

To adjust for differences in baseline characteristics (body mass index, creatinine, extracardiac arteriopathy, three vessel CAD, STEMI, history of stroke, arterial hypertension, reduced ejection fraction less than 35%), differences in procedure parameters (urgent procedure, off-pump surgery, aortic cross-clamp time [ACC] extracorporeal circulation time, procedure duration) and surgeon’s specifics (surgeon’s ID, annual surgery volume), outcome comparisons were performed using multivariate logistic regression.

Influence of annual surgeon CABG procedure volume on postoperative outcome was assessed by using RCS transformation of the annual surgeon procedure volume and integrated in the multivariate regression model. For the RCS analysis, we used 4 degrees of freedom (df = 4) and placed the knots at default quantiles as specified by the rcs() function in R. RCS curves for outcome parameters myocardial infarction, renal failure, stroke and mortality are shown in Supplementary Fig. S1.

Continuous variables are reported as medians with interquartile range (IQR; 25th percentile, 75th percentile) and compared using the Mann–Whitney U-test. Binary variables are shown as counts and frequencies and compared using the χ2 test.

Normality of continuous data deviation was tested by using the Shapiro–Wilk test.

Survival analysis was performed using log-rank test and displayed using a Kaplan–Meier curve.

For the cumulative incidences of non-fatal events, postoperative mortality was considered as competing events.

Competing risk analysis was performed using the Aalen-Johansen estimator to calculate cumulative incidence of stroke, myocardial infarction and renal failure, with mortality as the competing event.

Differences between the study groups were tested using the Gray’s test.

*P*-values were reported without correction for multiple testing. A level of significance was set to two-tailed *P *<* *0.05.

All statistical analyses were performed using the statistical software RStudio 2023.12.1 (R Foundation for Statistical Computing, Vienna, Austria).

## RESULTS

### Baseline demographics

From 2009 to 2021, a total of 4912 consecutive patients underwent isolated CABG at our institution. Of those, 2556 (52.0%) were provided with BIMA only grafting. Following a preoperative evaluation conducted by experienced CABG surgeons, a total of 2125 patients were allocated to group 1 (CABG performed by staff surgeons), and 431 patients were assigned to group 2 (CABG performed by residents under the supervision of staff surgeons).

Patients undergoing complete arterial myocardial revascularization performed by specialist cardiovascular surgeons (group 1) presented with pronounced differences in baseline characteristics compared to patients being operated by residents in cardiovascular surgery (group 2). Proportion of male sex and patient’s age were without significant difference between groups. Group 2 presented with a higher BMI (group 1: 27.4 [IQR 25.0–30.4] vs group 2: 27.7 [IQR 25.3–31.1] kg/m^2^; *P* = 0.048). Rates of common comorbidities were different with a higher prevalence of hypertension in group 2 (group 1: 79.2% vs group 2: 84.3%; *P* = 0.022) and more extracardiac arteriopathy in group 1 (group 1: 17.3% vs group 2: 11.1%; *P* = 0.002). Renal function represented by preoperative creatinine levels was more impaired in group 1 (group 1: 0.97 [IQR 0.85–1.10] vs group 2: 0.94 [IQR 0.82–1.10] mg/dl; *P* = 0.017). Significantly more patients in group 1 presented with an ejection fraction ≤35% (group 1: 5.0% vs group 2: 2.3%; *P* = 0.020) and three-vessel CAD (group 1: 76.5% vs group 2: 70.1%; *P* = 0.006). More patients in group 1 presented with a history of stroke (group 1: 7.9% vs group 2: 4.9%; *P* = 0.039). Overall, this resulted in a higher risk profile in group 1 as reflected by EuroSCORE II (group 1: 1.18 [IQR 0.87–1.73] vs group 2: 1.05 [IQR 0.80–1.44]; *P* < 0.001). More patients in group 1 presented with acute STEMI (group 1: 6.5% vs group 2: 2.8%; *P* = 0.004) and more often underwent CABG as urgent procedure (group 1: 6.6% vs group 2: 1.6%; *P* < 0.001). Detailed baseline characteristics are summarized in Table 1.

**Table 1:** Baseline characteristics

	Group 1 staff surgeon (*n* = 2125)	Group 2 resident (*n* = 431)	*P*-value
Male, *n* (%)	1840 (86.6)	369 (85.6)	0.645
Age [years], median (IQR)	65.8 (58.8–71.6)	64.7 (58.3–70.2)	0.056
BMI [kg/m^2^], median (IQR)	27.4 (25.0–30.4)	27.7 (25.3–31.1)	0.048
EuroSCORE II, median (IQR)	1.18 (0.87–1.73)	1.05 (0.80–1.44)	<0.001
Ejection fraction <35%, *n* (%)	107 (5.0)	10 (2.3)	0.020
Three-vessel CAD, *n* (%)	1626 (76.5)	302 (70.1)	0.006
Redo surgery, *n* (%)	13 (0.6)	0 (0.0)	0.209
Prior CABG, *n* (%)	13 (0.6)	0 (0.0)	0.209
Prior PCI, *n* (%)	484 (22.8)	98 (22.7)	1.000
STEMI, *n* (%)	138 (6.5)	12 (2.8)	0.004
NSTEMI, *n* (%)	414 (19.5)	85 (19.7)	0.962
Extracardiac arteriopathy, *n* (%)	368 (17.3)	48 (11.1)	0.002
COPD, *n* (%)	98 (4.6)	22 (5.1)	0.752
Prior stroke, *n* (%)	167 (7.9)	21 (4.9)	0.039
NYHA ≥ III, *n* (%)	975 (45.9)	193 (44.8)	0.714
Hypertension, *n* (%)	1684 (79.2)	363 (84.2)	0.022
IDDM, *n* (%)	59 (2.8)	15 (3.5)	0.524
Creatinine [mg/dl], median (IQR)	0.97 (0.85–1.10)	0.94 (0.82–1.10)	0.017
Dialysis, *n* (%)	23 (1.1)	2 (0.5)	0.357
Urgency, *n* (%)	141 (6.6)	7 (1.6)	<0.001

BMI: body mass index; EuroSCORE II: European System for Cardiac Operative Risk Evaluation; CAD: coronary artery disease; CABG: coronary artery bypass grafting; PCI: percutaneous coronary intervention; STEMI: ST-elevation myocardial infarction; NSTEMI: non ST-elevation myocardial infarction; COPD: chronic obstructive pulmonary disease; NYHA: New York Heart Association; IDDM: insulin-dependent diabetes mellitus.

To assess the learning-curve in the first CABG cases of each surgeon, a CUSUM analysis was performed and is shown in Supplementary Fig. S2. This analysis showed that none of the surgeons exhibited higher mortality rates than expected deviations over the upper CUSUM limit. These results suggest that none of the surgeons exhibited performance that would indicate a significant and repeated deviation in mortality rates. The mortality rates were generally within acceptable bounds, and no surgeon crossed a critical threshold that would suggest a systemic or significant issue. This may indicate that surgical quality remained stable and within expectations across all surgeons in the study. Total CABG cases per surgeon and annual procedure volume are shown in Supplementary Table S1.

### Procedural data

CABG in group 2 was more often performed as on-pump procedure (group 1: 52.1% vs group 2: 83.8%; *P* < 0.001) whereas the proportion of off-pump CABG was significantly higher in group 1 (group 1: 45.1% vs group 2: 15.3%; *P* < 0.001). The rate of conversion from off-pump to on-pump was more frequent in group 1 (group 1: 1.8% vs group 2: 0.2%; *P* = 0.026). Procedure duration was longer in group 2 (group 1: 255 [IQR 215–300] vs group 2: 280 [IQR 247–330] min.; *P* < 0.001), but not extracorporeal circulation (group 1: 103 [IQR 86–124] vs group 2: 107 [IQR 87–126] min.; *P* = 0.102) and ACC (group 1: 74 [IQR 60–91.5] vs group 2: 77 [IQR 62–95] min.; *P* = 0.107) (calculation for on-pump procedures only). Rate of sequential grafting (group 1: 52.5% vs group 2: 43.2%; *P* < 0.001) and the number of bypasses was lower in group 2 (*P* < 0.001), while complete revascularization was achieved more often in group 2 (group 1: 76.4% vs group 2: 82.6%; *P* = 0.006). More anastomoses in the right coronary artery territory were performed by staff surgeons (group 1: 52.7% vs group 2: 42.2%; *P* < 0.001). Detailed procedural data are summarized in Table 2.

**Table 2:** Procedural data

	Group 1 staff surgeon (*n* = 2125)	Group 2 resident (*n* = 431)	*P*-value
Type of CABG procedure			
On-pump arrest, *n* (%)	1107 (52.1)	361 (83.8)	<0.001
On-pump beating, *n* (%)	59 (2.8)	4 (0.9)	0.037
Off-pump, *n* (%)	959 (45.1)	66 (15.3)	<0.001
Conversion to on-pump, *n* (%)	39 (1.8)	1 (0.2)	0.026
Procedural times			
Procedure duration, median (IQR)	255 (215–300)	280 (247–330)	<0.001
ECC, median (IQR)^a^	103 (86–124)	107 (87–126)	0.102
ACC, median (IQR)^a^	74 (60–91.5)	77 (62–95)	0.107
Complete revascularization, *n* (%)	1624 (76.4)	356 (82.6)	0.006
Number of bypasses			
1, *n* (%)	3 (0.1)	0 (0.0)	<0.001
2, *n* (%)	1006 (47.3)	245 (56.8)	
3, *n* (%)	974 (45.8)	174 (40.4)	
4, *n* (%)	136 (6.4)	12 (2.8)	
5, *n* (%)	6 (0.3)	0 (0.0)	
Composite grafting, *n* (%)	1334 (62.8)	210 (48.7)	<0.001
Anastomosis RCA territory, *n* (%)	1120 (52.7)	182 (42.2)	<0.001
Anastomosis Cx territory, *n* (%)	668 (31.4)	138 (32.0)	0.857

a on-pump procedures only.

CABG: coronary artery bypass grafting; ECC: extracorporeal circulation time; ACC: aortic cross-clamp time; RCA: right coronary artery; Cx: circumflex artery.

### Postprocedural and clinical outcome data

After adjustment for differing baseline characteristics, annual surgeon CABG procedure volume and differences in procedural data, postprocedural parameters and outcome parameters during the first 30 days after CABG were without significant differences between groups. Postoperative use of mechanical circulatory support was not different between groups. Rate of prolonged ventilation and the need for catecholamine therapy for more than 24 hours postoperatively did not differ between groups (group 1: 2.3% vs group 2: 3.0%; *P* = 0.186 0.163; group 1: 1.5% vs group 2: 2.1%; *P* = 0.316). Rethoracotomy for bleeding (group 1: 3.2% vs group 2: 3.2%; *P* = 0.741), wound infection (group 1: 4.0% vs group 2: 3.9%; *P* = 0.265) and DSWI (group 1: 1.6% vs group 2: 1.6%; *P* = 0.299) showed no significant differences. Length of ICU and in-hospital stay were similar in both groups (group 1: 2 [IQR 1–3] vs group 2: 2 [IQR 1–3] days; *P* = 0.919; group 1: 7 [IQR 6–8] vs group 2: 7 [IQR 6–8] days; *P* = 0.291).

**Figure 2:**
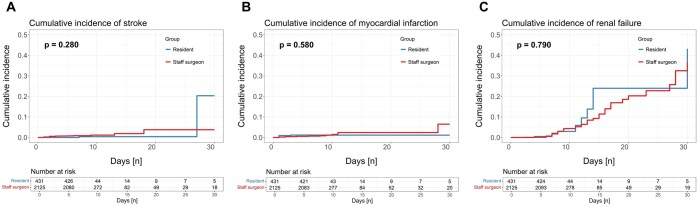
Cumulative incidences stratified by groups for primary outcome parameters (A) stroke, (B) myocardial infarction, and (C) renal failure. During the first 30 days after CABG procedure no significant differences in postoperative stroke (group 1: 1.1% vs. group 2: 0.7%; *P* = 0.280), myocardial infarction (group 1: 1.1% vs. group 2: 1.2%; *P* = 0.580), or renal failure (every KDIGO stadium) (group 1: 3.4% vs. group 2: 3.7%; *P* = 0.790) were determined between groups

**Figure 3:**
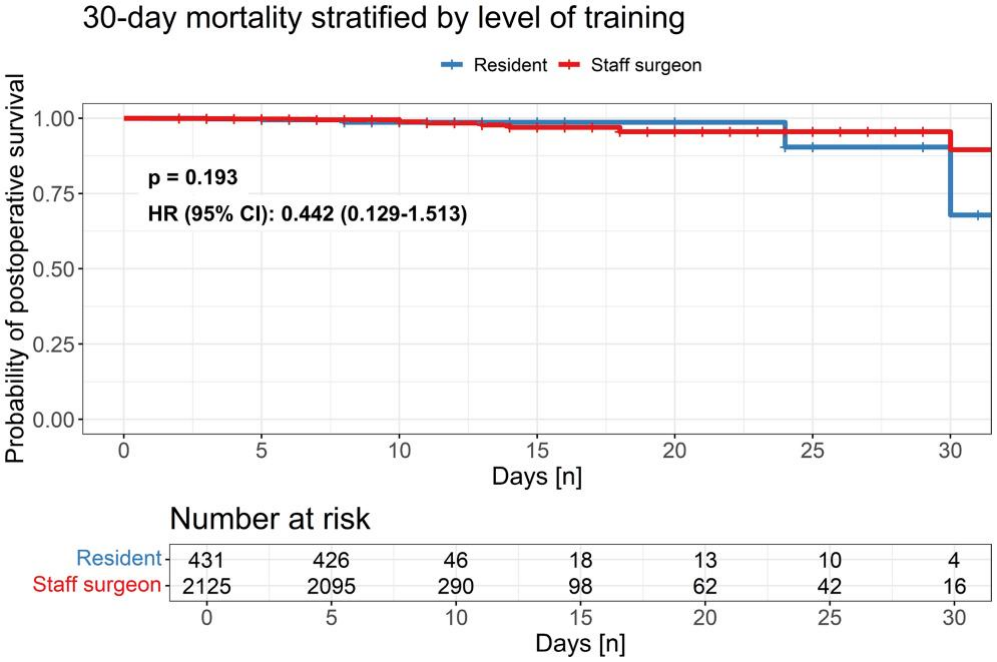
Thirty-day survival curves stratified by groups. During the first 30 days after CABG procedure no significant difference in all-cause mortality (group 1: 0.8% vs. group 2: 1.2%; HR (95% CI): 0.442 (0.129-1.513); *P* = 0.193) was determined between groups. HR: hazard ratio; CI: confidence interval

During the first 30 days after CABG no significant differences in all-cause mortality (group 1: 0.8% vs group 2: 1.2%; HR [95% CI]: 0.442 [0.129–1.513]; *P* = 0.193), postoperative myocardial infarction (group 1: 1.1% vs group 2: 1.2%; *P* = 0.580), stroke (group 1: 1.1% vs group 2: 0.7%; *P* = 0.280) or renal failure (every KDIGO stadium) (group 1: 3.4% vs group 2: 3.7%; *P* = 0.790) were determined between groups. Detailed postprocedural and clinical outcome data are summarized in Table 3. Cumulative incidences of primary outcome parameters are presented in Fig. 2. Survival curves are shown in Fig. 3.

**Table 3:** Postprocedural data and outcome parameter

	Group 1 staff surgeon (*n* = 2125)	Group 2 resident (*n* = 431)	Unadjusted *P*-value	Adjusted *P*-value
Postoperative MCS				
IABP, *n* (%)	15 (0.7)	1 (0.2)	0.422	0.398
ECMO, *n* (%)	5 (0.2)	2 (0.5)	0.747	0.398
Impella, *n* (%)	8 (0.4)	0 (0.0)	0.422	0.993
Prolonged ventilation > 24 h, *n* (%)	49 (2.3)	13 (3.0)	0.482	0.186
Need for catecholamines > 24 h, *n* (%)	31 (1.5)	9 (2.1)	0.455	0.316
Rethoracotomy due to bleeding, *n* (%)	67 (3.2)	14 (3.2)	1.000	0.741
Wound infection, *n* (%)	85 (4.0)	17 (3.9)	1.000	0.265
Deep sternal wound infection, *n* (%)	34 (1.6)	7 (1.6)	1.000	0.299
ICU stay [days], median (IQR)	2 (1–3)	2 (1–3)	1.000	0.919
Hospital stay [days], median (IQR)	7 (6–8)	7 (6–8)	1.000	0.291
30-day outcome				
Myocardial infarction, *n* (%)	24 (1.1)	5 (1.2)	1.000	0.580
Renal failure KDIGO ≥ 1, *n* (%)	72 (3.4)	16 (3.7)	0.848	0.790
Stroke, *n* (%)	24 (1.1)	3 (0.7)	0.586	0.280
Mortality, *n* (%)	16 (0.8)	5 (1.2)	0.575	0.193

MCS: mechanical circulatory support; IABP: intra-aortic balloon pump; ECMO: extracorporeal membrane oxygenation; h: hours; ICU: intensive care unit; KDIGO: Kidney Disease: Improving Global Outcomes.

## DISCUSSION

### Main findings

Main findings of the herein conducted study are (i) complex CABG utilizing two internal mammary arterial grafts can be safely performed by residents under supervision from the start of surgical training and provides reasonable patient outcomes, (ii) preoperative risk assessment conducted by an experienced cardiac surgeon specialized in CABG may be of crucial importance for adequate patient selection, and (iii) the herein introduced training concept for complex CABG training increases overall BIMA use at our centre omitting application of SVG in most cases and assures CABG guideline adherence independent from stage of surgical training.

Despite the fact that guidelines for myocardial revascularization recommend use of two arterial grafts for CABG in most patients, there is still a widespread use of SVG. The 2022 published SYNTAX Extended survival study revealed use of a single mammary artery in 68.3% of patients with a significant long-term survival benefit of patients undergoing CABG with application of two arterial grafts [13]. Survival benefit of total arterial revascularization was shown in multiple registry analyses in the past [4, 14], whereas long-term analysis of the Arterial Revascularization Trial (ART) revealed impact of surgeon experience on cardiovascular event rates after CABG [15]. Another factor which potentially influences long-term graft patency and long-term survival may be CABG technique in terms of utilization of cardiopulmonary bypass (on-pump) or off-pump techniques, which was not addressed by the herein conducted study. Furthermore, there are remarkable differences in rates of total arterial revascularization between countries [7, 8]. Reasons for utilization of SVG differ including concerns regarding DSWIs after BIMA harvesting [16]. Zhu *et al.* showed that risk for DSWI was as low as for single IMA use when grafts were harvested in a skeletonized fashion [17]. In our analysis, rate of DSWI was low, which is possibly due to skeletonized IMA harvesting.

Skeletonized IMA harvesting and CABG using BIMA demands advanced surgical skills. Numerous reports of simulated based surgical training exist and show that adaption and improvement of skills happen rather quickly [18–20]. Fann *et al.* showed that already a 4-hour simulation based surgical training improved surgical skills significantly [21]. For surgical training, it is of paramount importance to implement a structured training programme, including dry- and wet-labs. Furthermore, to identify needs and capabilities of trainees. Li *et al.* showed that coronary artery anastomosis training improved residents’ technique skills significantly with achieving the same results as specialized cardiovascular surgeons [22]. An early implementation of a structured dry-lab training programme, aimed at enhancing surgical skills, may contribute to safer patient outcomes.

We herein showed that outcomes after complex CABG performed by residents under supervision and staff surgeons are not different in terms of mortality, stroke, myocardial infarction or acute kidney injury (AKI). However, trainees presented with longer procedure times. A phenomenon which is known from previous studies [23, 24]. It is important to highlight that staff surgeons were more frequently involved in performing technically more challenging procedures like complex bypass anastomoses in terms of higher rates of sequential grafting techniques compared to residents.

A meta-analysis by Virk *et al.* including 52 966 patients confirmed these results and showed no increase in short- or mid-term mortality for patients operated by residents, but longer perfusion and cross-clamp times [25], which were not seen in our analysis. The longer procedure times in CABG performed by residents were not associated with increased rates of adverse short-term outcomes, which suggest that the quality of the anastomosis and the integrity of the grafts are crucial factors for favourable early outcomes after CABG.

To our knowledge, no reports exist comparing outcomes of CABG using complex arterial revascularization performed by residents or staff surgeons. Our data suggest that complex arterial revascularization with composite and sequential grafting performed by residents under supervision is safe and presents excellent clinical results. A crucial step for implementing complete arterial CABG in an early phase of cardiac surgery training without compromising patient safety may be patient selection. In particular, our data suggest that mainly lower-risk patients requiring less anastomoses were chosen for surgical training by preoperative stratification of severity of CAD by an experienced CABG surgeon.

### Study limitations

Due to the retrospective nature of the herein conducted study, limitations are inherent in the study design: patients were not randomized to a specific treatment; therefore, patient preselection with hidden confounders may apply. Due to absence of objective selection criteria, there may be a possible bias of the selection process in terms of absence of objective selection patterns. Since the herein presented results cover a rather large time frame, surgical education schemes with training in dry- and wet-lab settings changed over time at our institution which may influence results.

A learning curve-associated increase in postoperative complication rates may be present in the first CABG cases of each surgeon. We assessed this topic by performing a CUSUM analysis. It is important to note that some surgeons have a low case volume, while others were active only as staff surgeons, not as residents, or moved from other centres, during the study period. As a result, evaluating the first surgeries performed as either a resident or staff surgeon is not entirely precise within the scope of the study period.

The study’s reliance on subjective preoperative assessments limits the generalizability of its findings.

In our analysis, a complete case analysis was performed, excluding cases with missing data to avoid bias from incomplete information. We acknowledge that this approach may lead to attrition bias.

Prospective studies, ideally randomized trials, are needed to confirm the study’s findings. Additionally, future research should explore long-term outcomes and the scalability of residents’ training models across other centres.

## CONCLUSIONS

Results of complex CABG performed by residents under supervision showed no significant differences in short-term outcomes compared to specialist cardiovascular surgeons. However, residents performed fewer composite grafting and fewer anastomoses to the RCA territory. These results show that complex complete arterial revascularization can be safely performed by residents with excellent results at an experienced centre highlighting the importance of structured training programmes and appropriate patient selection. An early exposure of residents to complex state of the art arterial revascularization strategies is crucial for their training and the patient’s outcome.

## Supplementary Material

ivaf100_Supplementary_Data

## Data Availability

The raw data supporting the conclusions of this article will be made available by the authors, without undue reservation.

## ABBREVIATIONS

ACC: Aortic cross-clamp time
AKI: Acute kidney injury
BIMA: Bilateral internal mammary artery
CABG: Coronary artery bypass grafting
CAD: Coronary artery disease
DSWI: Deep sternal wound infection
ECC: Extracorporeal circulation time
ICU: Intensive care unit
IMA: Internal mammary artery
KDIGO: Kidney Disease: Improving Global Outcomes
LAD: Left anterior descending
LIMA: Left internal mammary artery
RA: Radial artery
RCS: Restricted cubic spline
RIMA: Right internal mammary artery
SVG: Saphenous vein graft
TTE: Transthoracic echocardiography
