# Supplementary material for: Coronary artery bypass grafting using both internal mammary arteries—a safe concept for surgical training
Source: Interdiscip Cardiovasc Thorac Surg. 2025 Apr 25;40(5):ivaf100. doi: 10.1093/icvts/ivaf100 (PMC12064215; doi:10.1093/icvts/ivaf100)
Supplement: ivaf100_Supplementary_Data [file ivaf100_supplementary_data.zip › CORRECT_supplemental_material.docx]

**SUPPLEMENTAL**

**Supplemental Table 1**: CABG cases per surgeon

| **Surgeon identification number** | **Total cases as resident** | **Total cases as staff surgeon** | **Cases per year** |
| --- | --- | --- | --- |
| 1 | 0 | 144 | 13.1 |
| 2 | 0 | 119 | 9.9 |
| 3 | 22 | 32 | 7.7 |
| 4 | 41 | 2 | 7.2 |
| 5 | 4 | 179 | 15.3 |
| 6 | 4 | 71 | 8.3 |
| 7 | 3 | 0 | 1.5 |
| 8 | 0 | 81 | 7.4 |
| 9 | 0 | 16 | 3.2 |
| 10 | 0 | 10 | 2.5 |
| 11 | 0 | 268 | 38.3 |
| 12 | 26 | 45 | 10.1 |
| 13 | 1 | 0 | 1.0 |
| 14 | 34 | 75 | 27.3 |
| 15 | 0 | 2 | 2.0 |
| 16 | 13 | 0 | 13.0 |
| 17 | 22 | 23 | 7.5 |
| 18 | 6 | 0 | 6.0 |
| 19 | 0 | 28 | 2.5 |
| 20 | 0 | 227 | 17.5 |
| 21 | 19 | 24 | 14.3 |
| 22 | 0 | 77 | 7.0 |
| 23 | 41 | 15 | 14.0 |
| 24 | 0 | 103 | 14.7 |
| 25 | 29 | 292 | 29.2 |
| 26 | 32 | 0 | 16.0 |
| 27 | 32 | 0 | 10.7 |
| 28 | 6 | 0 | 6.0 |
| 29 | 0 | 29 | 4.8 |
| 30 | 0 | 63 | 10.5 |
| 31 | 45 | 50 | 10.6 |
| 32 | 51 | 150 | 33.5 |

**Suppl. Fig. 1: Restricted cubic spline curves for 30-day outcome parameters myocardial infarction, stroke, renal failure and mortality.**

Restricted cubic spline analysis of annual surgeon procedure volume integrated in the multivariable regression model for outcome parameters.

**Suppl. Fig. 2: CUSUM analysis stratified by individual surgeon and devided in resident and staff surgeon groups.**

CUSUM analysis stratified by individual surgeon with CUSUM control limits set based on a 2-sigma approach (2 standard deviations from the expected mean).
