# Supplementary figures and images for: Coronary artery bypass grafting using both internal mammary arteries—a safe concept for surgical training
Source: Interdiscip Cardiovasc Thorac Surg. 2025 Apr 25;40(5):ivaf100. doi: 10.1093/icvts/ivaf100 (PMC12064215; doi:10.1093/icvts/ivaf100)

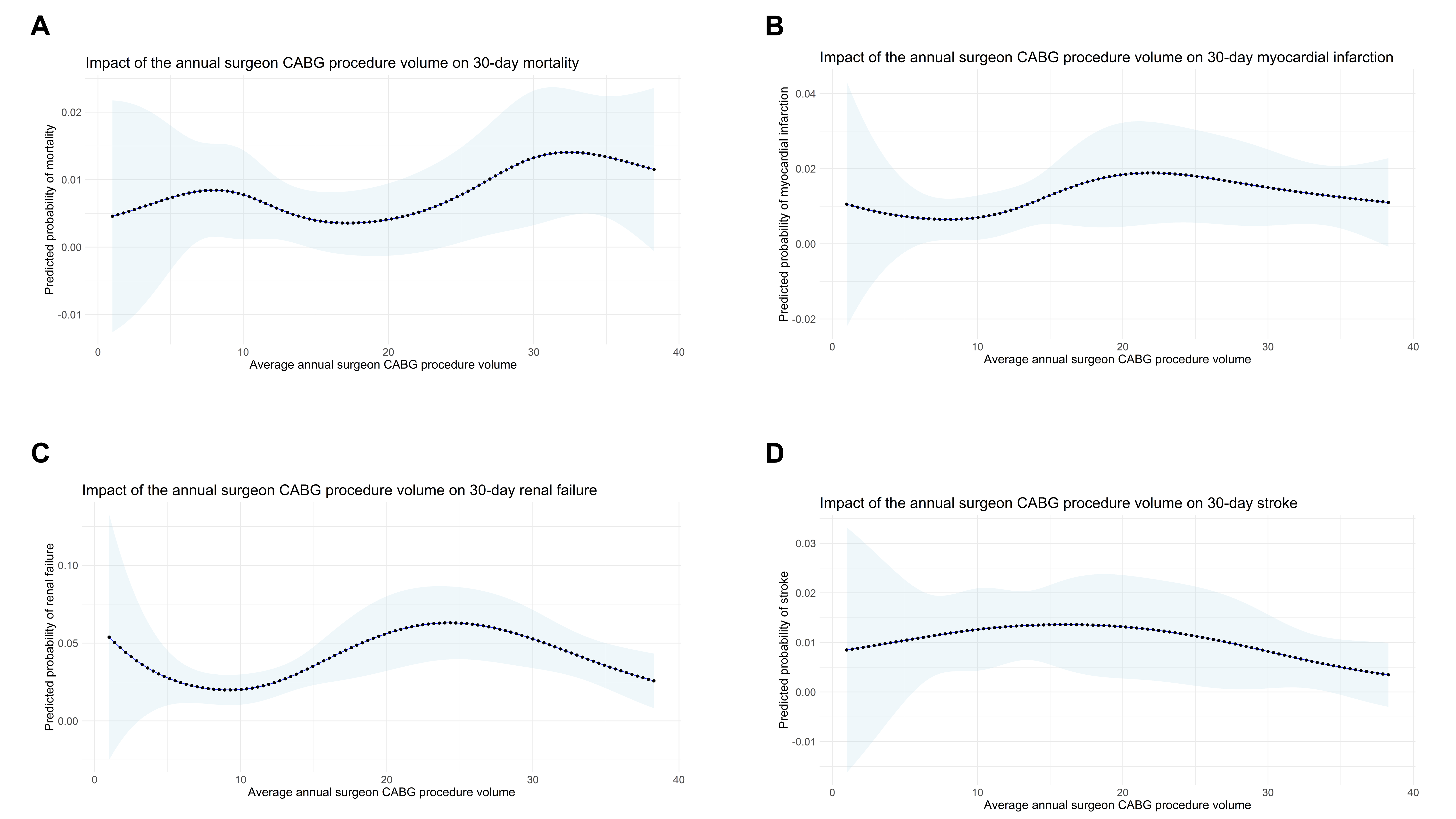

Supplement: ivaf100_Supplementary_Data [file ivaf100_supplementary_data.zip › Supp_Fig_1.tif]

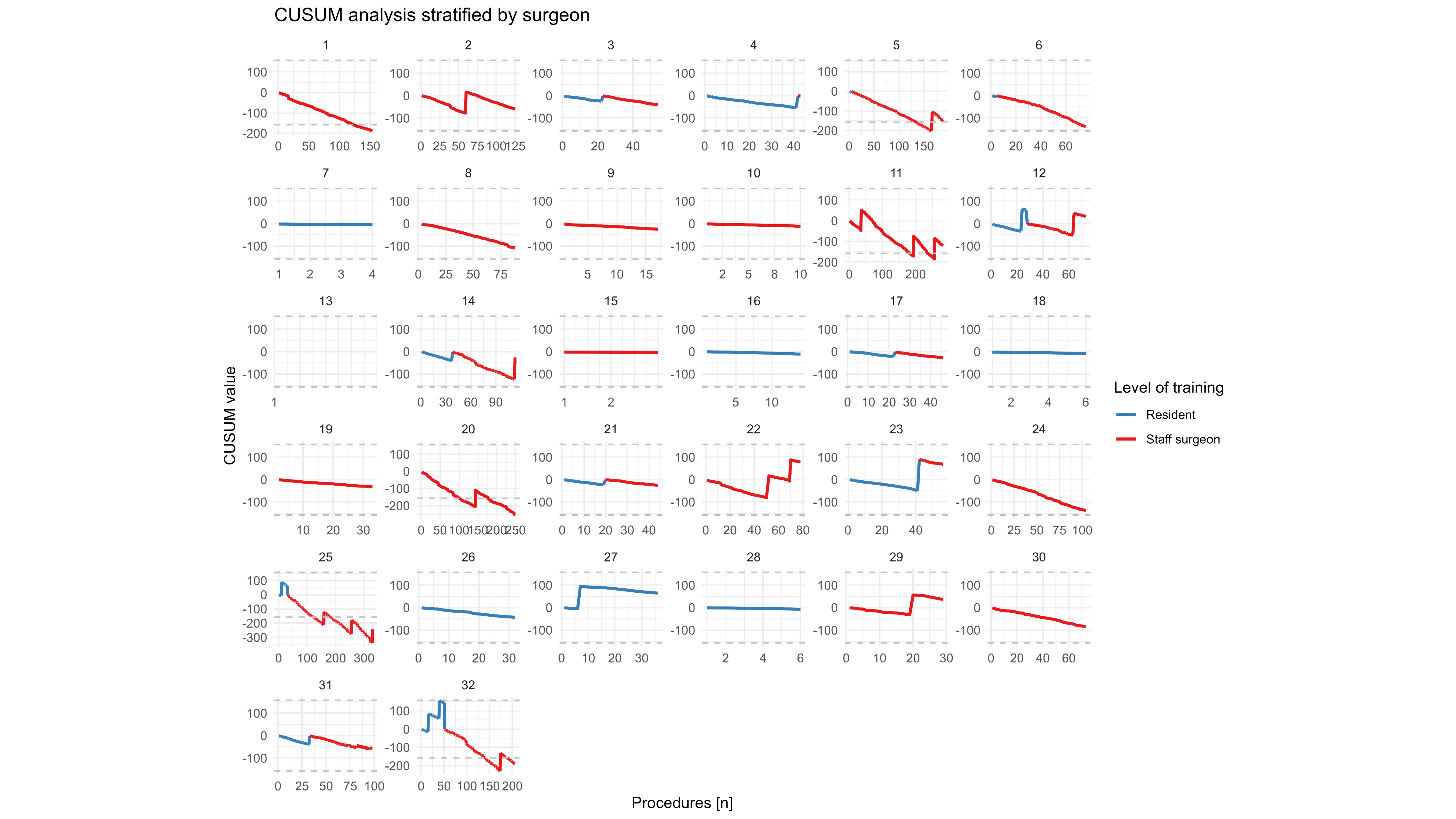

Supplement: ivaf100_Supplementary_Data [file ivaf100_supplementary_data.zip › Supp_Fig_2.tif]
